# Dietary supplementation with jasmine flower residue improves meat quality and flavor of goat

**DOI:** 10.3389/fnut.2023.1145841

**Published:** 2023-03-30

**Authors:** Jinxing Wang, Renhong Lu, Yehong Li, Junzhi Lu, Qiong Liang, Zihua Zheng, Heng Huang, Fuchang Deng, Huali Huang, Huimin Jiang, Junjie Hu, Ming Feng, Peng Xiao, Xiaogan Yang, Xingwei Liang, Jun Zeng

**Affiliations:** ^1^Guangxi Key Laboratory of Animal Breeding, Disease Control and Prevention, Guangxi University, Nanning, Guangxi, China; ^2^Institute for New Rural Development, Guangxi University, Nanning, China; ^3^Guangxi Nongken Yongxin Animal Husbandry Group Nasuo Animal Husbandry Co., Ltd., Nanning, China

**Keywords:** jasmine flower residue, meat quality, flavor, volatile component, goat meat

## Abstract

Jasmine flower residue (JFR) is a by-product retained in the production process of jasmine tea and can be used as an unconventional feed due to its rich nutrient value. This study aimed to evaluate the effects of the addition of JFR to the diet of goats on their meat quality and flavor. Twenty-four castrated Nubian male goats were randomly divided into two groups and fed a mixed diet containing 10% JFR (JFR, *n* = 12) or a conventional diet (CON, *n* = 12) for 45 days. Meat quality and flavor were measured at the end of the treatment. The addition of JFR to the diet could reduce the shear force of the longissimus dorsi muscle, as well as, the cross-sectional area and diameter of muscle fibers, indicating that the addition of JFR improved meat quality. JFR also increased the content of glutamic acid and ω-3 polyunsaturated fatty acid (C18:3n3 and C20:5N3) and reduced the content of C24:1 and saturated fatty acid (C20:0 and C22:0). In addition, the use of JFR increased the content of acetaldehyde and hexanal in the meat. Furthermore, JFR introduced new volatile components in the meat. The umami, saltiness, and richness of the meat also improved. In conclusion, the addition of jasmine flower residue to the diet can improve the meat quality and flavor of goat.

## 1. Introduction

Meat is a good source of protein, vitamins, minerals, essential polyunsaturated fatty acids, and other nutrients needed by the human body (1). Goat meat is valued for its advantages of tender meat, unique flavor, vitamins, and proteins, among others (2). As a main source of red meat, it has important effects on human health and nutrition. With the development of society and the economy, the demand for goat meat, as well as, for improved quality and flavor of meat has increased significantly (3).

Meat quality is evaluated based on various characteristics of meat, including pH, muscle color, water content, shear force, and other indicators that are closely related to the taste of meat (4, 5). Flavor is the sensory impression of smell and taste. Volatile components are the main source of cooked meat flavor and are obtained from a series of processes such as lipid oxidation and Maillard reaction during meat cooking (6). Meat quality and flavor are affected by animal breed, diet, gender, and other factors (7–11). In recent years, efforts have been made to improve goat meat quality and flavor by adjusting their diet structure and have shown success. Wang et al. conducted meat quality research and found that using the mixed silage of sweet sorghum and alfalfa in the goat diet can increase the subcutaneous fat thickness and water-holding capacity of mutton (12). Su et al. showed that the use of dietary alfalfa meals reduced the drip loss of mutton and increased the content of protein and fat (13). In addition, dietary structure adjustment has been shown to improve meat flavor. Hodges et al. reported that the use of corn-dried distiller grains instead of cottonseed meal or sorghum in the diet enhanced the umami flavor of mutton (14). Bianco et al. found that the addition of tannin extracts to the diet reduced the “pastoral” odor of fat (15).

Jasmine flower residue (JFR) is an unconventional feed that has not been studied much. It is a by-product of the jasmine tea industry and has not been effectively used for a long time due to the lack of economic benefits. The existing studies have shown that jasmine is rich in flavonoids and has anti-inflammatory, analgesic, and antipyretic effects (16). Moreover, Cheng et al. found that jasmine tea contained water-soluble polysaccharides with antioxidant activity and hypoglycemic effects (17). In addition, jasmine tea also has tranquilizing and anti-anxiety effects (18, 19).

To the best of our knowledge, to this date, the effect of JFR on the quality and flavor of goat meat has not been investigated. Therefore, the present study was conducted to investigate the effects of JFR addition to goat diets on the meat quality and flavor of goat.

## 2. Materials and methods

### 2.1. Animals and management

Twenty-four 4-month-old castrated Nubian male goats with similar body weight were randomly divided into two groups: (a) the CON group in which goats were fed a normal diet (*n* = 12) and (b) the JFR group in which goats were fed a diet containing 10% JFR (*n* = 12). The composition and nutrition levels of the diet are shown in Table 1. After an adaptation period of 7 days under the experimental conditions, the goats were fed the experimental treatments for 45 days. Water was always available to the animals.

**Table 1:** Composition and nutrient levels of diets (air-dry matter basis).

Item	Levels of inclusion of jasmine flower residues (%)	
	CON	JFR
Napier grass, (%)	25.00	25.00
Cassava alcohol residue, (%)	32.00	32.00
Corn, (%)	20.00	14.00
Soybean meal, (%)	9.00	5.00
Wheat bran, (%)	2.50	2.50
Barley, (%)	4.00	4.00
Jasmine flower residues, (%)	0.00	10.00
Sodium chloride, (%)	0.75	0.75
Calcium hydrogen phosphate, (%)	0.50	0.50
Mineral/vitamin premix, (%)	5.00	5.00
Limestone, (%)	1.00	1.00
Natrium Bicarbonate, (%)	0.25	0.25
Total	100.00	100.00
**Nutritional level**		
Metabolizable energy. ME (kcal/kg DM)	2.36	2.21
Crude protein, (%)	18.90	18.00
Ether extract, EE, (%)	2.80	2.70
Neutral detergent fiber, NDF, (%)	40.23	43.19
Acid detergent fiber, ADF, (%)	23.60	28.40
Calcium, (%)	1.26	1.25
Phosphorus, (%)	0.44	0.54
Lignin (%)	2.00	2.80

JFR was purchased from Guangxi hengxian jasmine flower factory (Nanning, China). The diet containing 10% JFR had the same nutrient content as the original diet.

### 2.2. Sample collection

At the end of the experiment, the goats were weighed and sacrificed by carotid artery exsanguination. All goats were stopped from feeding and drinking 12 h before sacrifice. After slaughter, take the longest dorsal muscle from the beginning of the thoracic spine to the end of the lumbar spine. The muscle located between the last and penultimate rib on the left longissimus dorsi muscle was used for flavor analysis, and the rest was used to evaluate meat quality. Samples for flavor analysis and the right longissimus dorsi muscle were treated with liquid nitrogen and stored at −80°C until used for quantitative analysis.

### 2.3. Meat quality analysis

Characteristics of the meat, namely, pH, muscle color, water loss rate, cooking loss, and shear force, were measured. pH was measured at 1 and 24 h postmortem using a pH meter (pH-STAR, MATTHAUS, Germany). Other indicators were measured 1 h after slaughter. A portion of the sample was weighed, put into an oven, and baked for 4 h at 105°C, after which it was weighed again to calculate the moisture content. Muscle color, which included lightness (L^*^), redness (a^*^), and yellowness (b^*^), was measured using a colorimeter (NR20XE, Shenzhen Sanenchi Technology Co., Ltd., China). A portion of the samples was weighed, heated in water at 80°C for 20 min, and weighed again to calculate the cooking loss. Following the method described by Rossi et al. (20), the shearing force was measured using a muscle shearing apparatus (RH-N50, Guangzhou Runhu Instrument Co., Ltd., China). To measure the water loss rate, the samples were wrapped with filter paper and hard plates, placed on the platform of the meat department hydraulic analyzer (RH-1000, Guangzhou Runhu Instruments Co., Ltd., China), pressurized to 35 kg for 5 min, and weighed again to calculate the water loss rate.

### 2.4. Muscle fiber characteristics analysis

The muscle was embedded in paraffin and cut into 5 μm sections. After dehydration, the sections were stained with hematoxylin and eosin (HE staining) and observed under a microscope (TCS-SP8MP, Leica). Three areas in the slide were randomly selected and the cross-sectional area, diameter, and density of the muscle fibers were examined using Image-Pro Plus (version 6.0, Media Cybernetics, United States).

### 2.5. Amino acid detection

The amino acid sample solution was prepared according to GB5009.124-2016, and equal volumes of the sample and standard solutions were injected into an amino acid analyzer (LA8080, Hitachi, Japan) to calculate amino acid concentration.

### 2.6. Fatty acid detection

The extraction and methylation of fatty acids in samples were conducted about the method of Banco (21). The liquid obtained from the extraction was filtered through a 0.45 μm membrane filter and tested using gas chromatography–mass spectrometry (Trace1310, Thermo, United States). The GC/MS program was as follows. Chromatographic column: TG-5MS (30 m × 0.25 mm × 0.25 μm). Temperature programming: 80°C was maintained for 1 min, then increased to 200°C at the rate of 10°C/min, then to 250°C at the rate of 5°C/min, and finally increased to 270°C at the rate of 2°C/min for 3 min; inlet temperature: 290°C. Flow rate of the carrier gas: 1.2 ml/min; split ratio: no split; ion source temperature: 280°C; transmission line temperature: 280°C; solvent delay time: 5.00 min; scanning range: 30–400 amu; energy of ionization: 70 eV.

### 2.7. Analysis of volatile compounds by HS-SPME/GC–MS

The samples were placed in a headspace vial, sealed with 3 ml of saturated NaCl solution, and equilibrated at 80°C for 30 min. The solid-phase microextraction needle (SUPELCO, United States) was used for extraction for 30 min. After the extraction, the needle was placed at the injection port for desorption for 5 min. The volatile compounds were separated and identified using gas chromatography–mass spectrometry (GC–MS, 6890 N-5975B, Agilent, United States) (22). The collected mass spectra were retrieved using the NIST database to identify the volatile components in the samples, and the relative contents of each component were analyzed by area normalization. The operating conditions of the GC/MS instrument were as follows. Chromatographic column: HP-5MS (30 m × 0.25 mm × 0.25 μm); split ratio: no split; carrier gas flow rate: 1.0 ml/min; inlet temperature: 240°C; scan method: full scan. Temperature of ion source: 230°C; four-stage rod temperature: 180°C. Temperature programming: initial temperature maintained at 45°C for 4 min, increased to 130°C at a rate of 6°C/min, and then increased to 240°C at a rate of 10°C/min for 8 min.

### 2.8. Olfactory characteristics determination by electronic nose

The electronic nose system contained 10 sensors that were sensitive to different types of volatile substances: W1C (Benzene), W5S (Nitrogen oxides), W3C (ammonia), W6S (hydrogen), W5C (alkane), W1S (Short-chain alkanes), W1W (Inorganic sulfide), W2S (Alcohols, ethers, aldehydes, ketones), W2W (Organic sulfide), and W3S (Long-chain alkanes). First, the two groups of samples were thawed at 4°C. Then, 10 g of samples were weighed and placed in a 100 ml beaker, sealed with double-layer cling film, and allowed to stand for 1 h at room temperature. The injection needle was inserted into a sealed beaker containing the sample, and volatile substances were detected by an electronic nose system (PEN3, AIRSENSE, Germany). Determination conditions: sampling time was 1 s/group, sensor self-cleaning time was 80 s, and the sample preparation time was 5 s. The injection flow rate was 400 ml/min. The waiting time for analysis was 80 s.

### 2.9. Taste characteristics determination by electronic tongue

The electronic tongue system (TS-5000Z, INSENT Company, Japan) was used to detect the taste characteristics of the two groups of samples. Nine flavors were detected by nine sensors. First, the two groups of samples were thawed at 4°C, and 40 g of samples were weighed. To this, 200 ml of purified water was added and the mixture was poured into a domestic cooking machine and stirred for 30 s for uniform mixing. The samples were then poured into a centrifuge tube and centrifuged at 3,000 rpm for 5 min; 80 ml of supernatant was collected and tested using a taste analysis system. Analytical conditions: The potential of the reference solution (30 mM potassium chloride +0.3 mM tartaric acid) was used as the reference potential. The probe was cleaned with the sample solution for 20 s and then immersed into the sample solution to measure its potential. Finally, the result was calculated by the system.

### 2.10. Statistical analysis

Statistical analysis was performed using IBM SPSS Statistics Version 23.0.0 (IBM Incorporation, United States) and Microsoft Office Excel 2019 (Microsoft Corporation, USA). Data were analyzed using Student’s *t*-test and expressed as means ± SEMs. *p* < 0.05 was considered statistically significant.

## 3. Results

### 3.1. Effect of JFR on meat quality

To evaluate the effect of JFR on goat meat quality, pH (pH_1h_ and pH_24h_), muscle color (L*, a*, and b*), moisture content, water loss rate, and shear force of the longissimus dorsi muscle of the two groups were evaluated (Table 2). The results showed that compared with the CON group, the shearing force of the JFR group was significantly reduced (*p* < 0.05), while there were no significant differences in the pH, muscle color, water content, and water loss rate (*p* > 0.05). This result indicated that JFR could improve the tenderness of goat meat.

**Table 2:** Effect of dietary jasmine flower residue supplementation on meat quality.

Items	Treatments		*p* value
	CON	JFR	
**pH**			
pH_1h_	6.74 ± 0.04	6.60 ± 0.06	0.065
pH_24h_	5.92 ± 0.04	6.08 ± 0.09	0.15
**Color**			
Lightness (L^*^)	37.24 ± 0.52	36.13 ± 0.67	0.217
Redness (a^*^)	15.13 ± 0.26	15.34 ± 0.36	0.645
Yellowness (b^*^)	4.53 ± 0.07	4.22 ± 0.17	0.121
Moisture content (%)	73.87 ± 0.26	72.82 ± 0.56	0.121
Water loss rate (%)	16.21 ± 1.09	19.01 ± 1.08	0.097
Cooking loss (%)	14.73 ± 2.31	13.74 ± 0.61	0.689
Shear force (N)	85.06 ± 3.92^a^	69.41 ± 4.51^b^	0.025

^a,b^The values within a row with different superscripts are significantly different (*p* < 0.05).

### 3.2. Effect of JFR on muscle fiber characteristics

**Figure 1:**
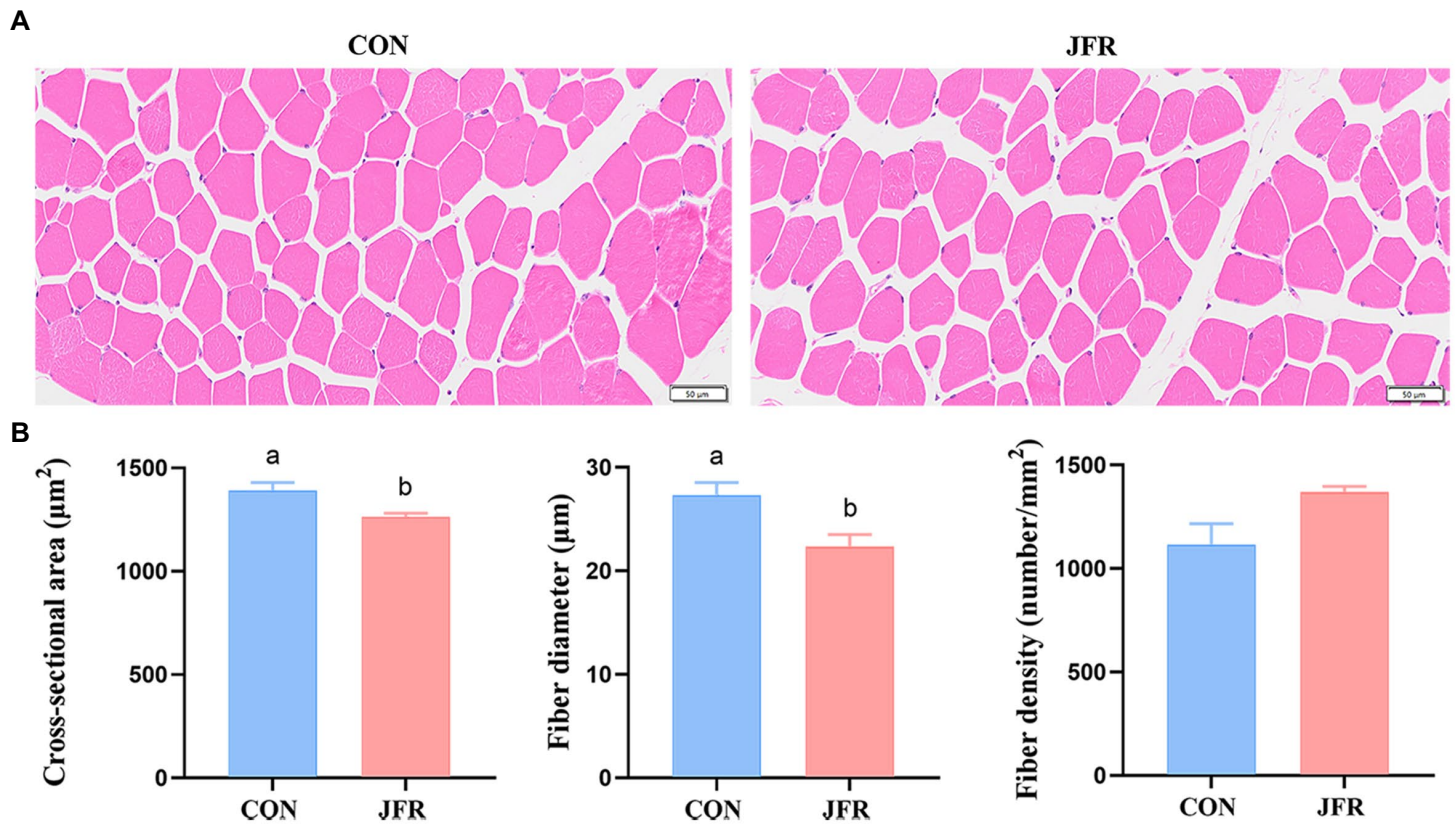
Effect of JFR on muscle fiber characteristics. **(A)** Hematoxylin and eosin staining of longissimus dorsi muscle (Bar = 50 μm). **(B)** Statistical results of cross-sectional area, diameter and density of muscle fibers. The values with different letters (a and b) are significantly different (*p* < 0.05).

To further evaluate the effect of JFR on meat tenderness, we compared the muscle fiber characteristics of the two groups. As shown in Figures 1A,B, the cross-sectional area and diameter of muscle fibers in the JFR group were significantly smaller than those in the CON group (*p* < 0.05), while the density of muscle fibers in the JFR group was not significantly different from that in the CON group (*p* > 0.05).

### 3.3. Effect of JFR on amino acids, fatty acids, and volatile compounds

To explore the effect of changes in chemical composition on the taste and flavor of goat meat, the differences in amino acids, fatty acids, and volatile compounds in the two groups were compared. The differences in amino acids are shown in Table 3. The contents of 16 amino acids were different between the two groups. Compared with the CON group, glutamic acid (Glu) content in the JFR group was significantly increased (*p* < 0.05), while the contents of other amino acids were not significantly different (*p* > 0.05) between the two groups. The contents of total essential amino acids and delicious amino acids were significantly higher in the JFR group than in the CON group (*p* < 0.05), while there was no significant difference in the essential amino acids and total umami amino acids between the two groups (*p* > 0.05). In addition, there were no significant differences between the two groups in the ratio of essential amino acids to total amino acids (EAA/TAA), the ratio of non-essential amino acids to total amino acids (NEAA/TAA), and the ratio of essential amino acids to non-essential amino acids (EAA/NEAA; *p* > 0.05).

**Table 3:** Effect of dietary jasmine flower residue supplementation on the amino acid composition.

Items	Treatments		*p*-value
	CON	JFR	
Asp	1.87 ± 0.02	1.92 ± 0.02	0.176
Thr	0.94 ± 0.01	0.96 ± 0.02	0.240
Ser	0.78 ± 0.01	0.80 ± 0.02	0.350
Glu	3.12 ± 0.03^b^	3.26 ± 0.05^a^	0.036
Pro	0.76 ± 0.01	0.76 ± 0.01	0.702
Gly	0.94 ± 0.02	0.92 ± 0.01	0.558
Ala	1.18 ± 0.01	1.19 ± 0.01	0.605
Arg	1.32 ± 0.01	1.34 ± 0.01	0.208
Val	1.03 ± 0.02	1.05 ± 0.01	0.202
Met	0.18 ± 0.03	0.30 ± 0.07	0.109
Ile	0.95 ± 0.01	0.98 ± 0.01	0.213
Leu	1.68 ± 0.02	1.72 ± 0.02	0.142
Tyr	0.63 ± 0.01	0.65 ± 0.01	0.116
Phe	0.78 ± 0.01	0.80 ± 0.01	0.370
Lys	1.86 ± 0.02	1.91 ± 0.02	0.153
His	0.73 ± 0.02	0.76 ± 0.01	0.163
TAA	18.54 ± 0.26^b^	19.46 ± 0.28^a^	0.042
EAA	7.92 ± 0.11^b^	8.22 ± 0.08^a^	0.050
NEAA	10.77 ± 0.15	11.13 ± 0.18	0.161
FAA	8.50 ± 0.11	8.74 ± 0.11	0.162
EAA/TAA	0.43 ± 0.002	0.43 ± 0.002	0.600
NEAA/TAA	0.57 ± 0.002	0.58 ± 0.002	0.580
EAA/NEAA	0.73 ± 0.01	0.74 ± 0.01	0.650

EAA: essential amino acid (Thr + Val + Ile + Leu + Phe + Lys + His). NEAA: nonessential amino acid (Asp + Ser + Glu + Pro + Gly + Ala + Arg + Met + Tyr). FAA: delicious amino acids (Asp + Glu + Gly + Ala + Tyr + Phe). ^a,b^The values within a row with different superscripts are significantly different (*p* < 0.05).

As shown in Table 4, a total of 25 fatty acids, including 11 saturated fatty acids, 5 monounsaturated fatty acids, and 9 polyunsaturated fatty acids, were detected in the two groups. Compared with the CON group, the contents of C20:0 and C22:0 were decreased significantly (*p* < 0.05) in the saturated fatty acids (SFAs) of the JFR group. In the case of unsaturated fatty acids (UFAs), the content of C24:1 was significantly reduced (*p* < 0.05), and that of C18:3n3 (ALA) and C20:5 N3 (EPA) was significantly increased (*p* < 0.05) in the JFR than in the CON group. The contents of SFA, monounsaturated fatty acids (MUFAs), polyunsaturated fatty acids (PUFAs), and ω-6PUFA were not significantly different between the two groups (*p* > 0.05). However, compared with the CON group, ω-3PUFA was significantly increased (*p* < 0.05), and the ratio of ω-6PUFA to ω-3PUFA was significantly decreased (*p* < 0.05) in the JFR group. The results showed that JFR could change the composition of fatty acids, increase the content of ω-3PUFA and improve the nutritional value of goat meat.

**Table 4:** Effect of dietary jasmine flower residue supplementation on the fatty acid composition.

Items	Treatments		*p*-value
	CON	JFR	
C10:0	0.05 ± 0.01	0.05 ± 0.005	0.709
C12:0	0.08 ± 0.02	0.06 ± 0.01	0.129
C14:0	1.34 ± 0.16	1.14 ± 0.07	0.267
C15:0	0.207 ± 0.02	0.19 ± 0.01	0.419
C16:0	22.10 ± 0.38	20.87 ± 0.45	0.058
C17:0	0.93 ± 0.04	0.94 ± 0.05	0.822
C18:0	17.66 ± 0.95	17.02 ± 0.33	0.541
C20:0	0.08 ± 0.005^a^	0.06 ± 0.004^b^	0.041
C22:0	0.08 ± 0.01^a^	0.05 ± 0.01^b^	0.035
C23:0	0.06 ± 0.01	0.05 ± 0.01	0.224
C24:0	0.13 ± 0.01	0.11 ± 0.01	0.295
C14:1	0.07 ± 0.001	0.06 ± 0.001	0.433
C16:1	1.07 ± 010	1.08 ± 0.06	0.959
C18:1 N9C	43.81 ± 1.19	46.26 ± 1.15	0.165
C20:1	0.11 ± 0.01	0.11 ± 0.01	0.820
C24:1	0.07 ± 0.01^a^	0.04 ± 0.01^b^	0.045
C18:2n6c	7.34 ± 0.66	7.05 ± 0.60	0.723
C18:3n3	0.22 ± 0.02^b^	0.48 ± 0.05^a^	<0.001
C18:3n6	0.08 ± 0.01	0.08 ± 0.01	0.874
C20:2	0.08 ± 0.01	0.09 ± 0.01	0.227
C20:3n6	0.23 ± 0.02	0.20 ± 0.03	0.374
C20:4 N6	3.64 ± 0.42	3.42 ± 0.45	0.726
C20:5 N3	0.10 ± 0.01^b^	0.15 ± 0.02^a^	0.046
C22:1 N9	0.39 ± 0.06	0.36 ± 0.05	0.757
C22:6 N3	0.05 ± 0.002	0.06 ± 0.01	0.125
SFA	42.72 ± 0.956	40.55 ± 0.689	0.090
MUFA	45.13 ± 1.23	47.55 ± 1.15	0.175
PUFA	12.15 ± 1.13	11.90 ± 1.13	0.876
ω-6PUFA	11.32 ± 1.05	10.75 ± 1.02	0.701
ω-3 PUFA	0.37 ± 0.03^b^	0.70 ± 0.07^a^	0.001
ω-6/ω-3	30.87 ± 1.62^a^	15.57 ± 0.88^b^	<0.001

SFA: saturated fatty acids (C10:0 + C12:0 + C14:0 + C15:0 + C16:0 + C17:0 + C18:0 + C20:0 + C22:0 + C23:0 + C24:0). MUFA: monounsaturated fatty acids (C14:1 + C16:1 + C18:1N9C + C20:1 + C24:1). PUFA: polyunsaturated fatty acids (C18:2n6c + C18:3n3 + C18:3n6 + C20:2 + C20:3n6 + C20:4N6 + C20:5N3 + C22:1N9 + C22:6N3). ω-6PUFA: (C18:2n6c + C18:3n6 + C20:3n6 + C20:4N6). ω-3PUFA: (C18:3 n3 + C20:5N3 + C22:6N3). ^a,b^The values within a row with different superscripts are significantly different (*p* < 0.05).

A total of 125 volatile substances were detected in the two groups, of which 24 were available for analysis (the remaining could not be analyzed due to the small sample size detected) and included eight aldehydes, three alcohols, two ketones, five hydrocarbons, one furan, and five other substances (Table 5). Compared with the CON group, the contents of acetaldehyde and hexanal were significantly higher (*p* < 0.05) in the JFR group, and those of pentanal, isovaleraldehyde, heptanal, octanal, and nonanal in the two groups were not significantly different (*p* > 0.05) between the two groups. Isovaleraldehyde was detected in the CON but not in the JFR group. As for alcohols, 1-Octanol was only detected in the CON group, while pentanol and 1-Octen-3-OL were only detected in the JFR group. As for ketones, acetone was detected in both groups and there was no significant difference between them (*p* > 0.05), while cyclobutanone was only detected in the JFR group. As for hydrocarbons, ethylene oxide was detected in both groups and there was no significant difference between them (*p* > 0.05). Hexane, ethyl acetate, fluoroacetylene, and chlorobenzene were only detected in the JFR group. As for furans, 2-pentylfuran was detected in the JFR but not in the CON group. Finally, propionamide, cyanoacetamide, N-methyl-1,3-propanediamine, and N-methyl-1-octadecylamine were detected in both groups and there was no significant difference (*p* > 0.05), while dimethylhexylamine was only detected in the JFR group. The above results indicated that JFR could affect the flavor of goat meat by changing the composition of volatile components.

**Table 5:** Effect of dietary jasmine flower residue supplementation on volatile composition.

Items	Treatments		*p*-value
	CON	JFR	
**Aldehydes**			
Acetaldehyde	0.56 ± 0.15^b^	1.68 ± 0.40^a^	0.048
Pentanal	0.87 ± 0.72	2.45 ± 0.52	0.215
Isovaleraldehyde	0.93 ± 0.51	ND	
Hexanal	9.70 ± 4.28^b^	31.72 ± 6.58^a^	0.020
Benzaldehyde	1.47 ± 0.26	2.16 ± 0.25	0.126
Heptanal	4.63 ± 1.63	7.37 ± 0.96	0.172
Octanal	4.23 ± 1.71	4.64 ± 0.37	0.866
Nonanal	14.83 ± 2.62	15.20 ± 3.14	0.931
**Alcohols**			
1-Pentanol	ND	0.98 ± 0.04	
1-Octen-3-OL	ND	2.53 ± 1.03	
1-Octanol	1.40 ± 0.32	ND	
**Ketone**			
Acetone	1.10 ± 0.66	1.51 ± 0.05	0.662
Cyclobutanone	ND	0.39 ± 0.10	
**Hydrocarbon**			
Hexyl hydride	ND	3.09 ± 4.57	
Ethyl acetate	ND	2.55 ± 2.06	
Fluorine acetylene	ND	0.52 ± 0.13	
Ethylene Oxide	2.42 ± 1.15	2.09 ± 0.93	0.843
Chlorobenzene	ND	3.69 ± 1.12	
**Furan**			
2-Pentylfuran	ND	0.98 ± 0.38	
Anethole	66.69 ± 0.26	ND	
**Others**			
Propionamide	0.43 ± 0.06	0.42 ± 0.13	0.972
Cyanoacetamide	0.47 ± 0.18	0.39 ± 0.08	0.630
N-Methyl-1,3-propanediamine	0.38 ± 0.10	0.49 ± 0.06	0.316
Octodrine	ND	2.20 ± 1.46	
N-Methyl-n-octadecylamine	1.12 ± 0.52	1.12 ± 0.47	0.997

^a,b^The values within a row with different superscripts are significantly different (*p* < 0.05). ND: Not detected.

### 3.4. Effect of JFR on olfactory characteristics

**Figure 2:**
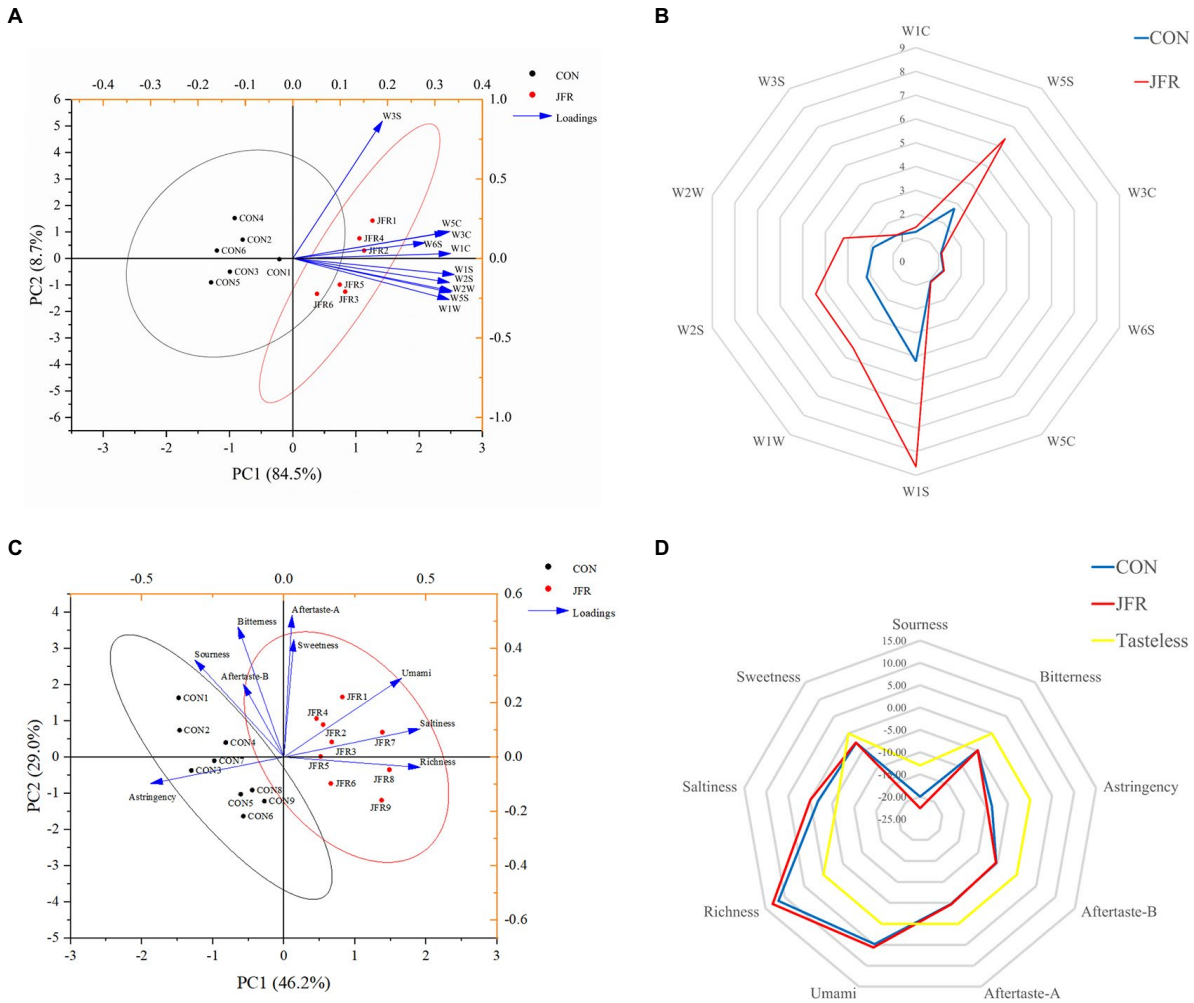
Effects of JFR on olfactory and taste characteristics. **(A)** Principal component analysis score map of goat meat aroma components based on electronic nose data. **(B)** Odor fingerprint of goat meat. The electronic nose system contained 10 sensors that were sensitive to different types of volatile substances: W1C (Benzene), W5S (Nitrogen oxides), W3C (ammonia), W6S (hydrogen), W5C (alkane), W1S (Short-chain alkanes), W1W (Inorganic sulfide), W2S (Alcohols, ethers, aldehydes, ketones), W2W (Organic sulfide), and W3S (Long-chain alkanes). **(C)** Principal component analysis score map of goat meat taste based on electronic tongue data. **(D)** Taste fingerprint of goat meat.

Figure 2A shows principal component (PC) analysis results for the spatial distribution and distance of odor for the two groups. The contribution rates of PC1 and PC2 to the total variance were 84.5 and 8.7%, respectively, indicating that the principal component could reflect the characteristics of the volatile gases of the two groups. The contribution of the sensors was then analyzed. The samples in the CON group mainly clustered near the negative axis of PC1 and correlated with W3S (Long-chain alkanes). The JFR group samples mainly clustered near the positive axis of PC1 and correlated with W1C (Benzene), W5S (Nitrogen oxides), W3C (ammonia), W6S (hydrogen), W5C (alkane), W1S (Short-chain alkanes), W1W (Inorganic sulfide), W2S (Alcohols, ethers, aldehydes, ketones), and W2W (Organic sulfide). These results suggest that the sensors of the electronic nose can distinguish two groups of samples effectively.

Figure 2B shows fingerprints of volatile fragrance material from two groups of samples based on electronic nose analysis results. The results showed that compared with the CON group, the meat of the JFR group had a more prominent odor and showed significantly increased (*p* < 0.05) aromatic components such as nitrogen oxides (W5S), short-chain alkanes (W1S), inorganic sulfide (W1W), alcohol, ether, aldehyde and ketone (W2S), and organic sulfide (W2W).

### 3.5. Effect of JFR on taste characteristics

Figure 2C shows principal component analysis results for the spatial distribution and distance of taste for the two groups. The contribution rates of PC1 and PC2 to the total variance were 46.2 and 29.0%, respectively, indicating that the principal component could reflect the taste characteristics of the two groups. The JFR group samples clustered near the positive axis of PC1 and correlated with sweetness, saltiness, richness, umami, and aftertaste-astringency. The CON group samples clustered near the negative axis of PC1 and correlated with astringency, sourness, bitterness, and aftertaste-bitterness. These results suggest that the electronic tongue sensor could effectively distinguish the two groups of samples.

Figure 2D shows a taste fingerprint based on the results of the electronic tongue taste test. Compared with the CON group, the sourness, bitterness, aftertaste-bitterness, and aftertaste-astringency were significantly reduced (*p* < 0.05), and the umami and richness were significantly increased (*p* < 0.05) in the JFR group. There was no significant effect on astringency, saltiness, and sweetness (*p* > 0.05). In addition, the saltiness and richness were higher than the tasteless point in both groups of samples. The sourness, bitterness, astringency, sweetness, aftertaste-bitterness, and aftertaste-astringency were lower than the tasteless point, indicating that these flavors were not shown in the two groups of goat meat samples. The results show that JFR can improve the taste of goat meat.

## 4. Discussion

The efficient use of unconventional feed can reduce feeding costs and improve economic efficiency. In recent years, the application of unconventional feed in goat breeding has gradually increased. For example, spinless cactus can be used in the place of hay to increase the carcass fat of lamb (23). Supplementing sea buckthorn pomace in the diet can improve the tenderness and water holding capacity of lamb meat (24). Bianco et al. found that adding cardoon meal to the diet could reduce the “animal” odor of fat and improve the flavor of lamb (25). In our study, the addition of JFR to the diet of goats reduced the shear force of the longissimus dorsi muscle and increased the content of glutamic acid and ω-3PUFA. Moreover, the content and types of volatile substances increased, and the umami and richness of meat also improved in JFR-fed goat meat.

Meat quality is closely related to taste. We found that the addition of JFR to the goat diet did not change the pH, water content, muscle color, and other indicators, but significantly reduced the shear force of the longest muscle. The characteristics of muscle fibers are also important factors affecting meat quality (26). The characteristics of muscle fibers, namely, the area and diameter of muscle fibers are positively related to the shearing force (27). We found that the addition of JFR to the diet reduced the cross-sectional area and diameter of muscle fibers, and may have directly contributed to decreasing the shear force. Among the muscle fiber types, the slow-twitch fiber was thinner than the fast-twitch fiber, and plant polyphenols could regulate the muscle fiber type by increasing the AMPK phosphorylation in the skeletal muscle and activating PGC-1α pathway (28, 29). Some animal experiments have shown that the addition of plant polyphenols such as apple polyphenols, proanthocyanidins and resveratrol in the diet can increase the proportion of slow-twitch fiber (30–32). Jasmine is rich in flavonoids, one of plant polyphenols (16). We speculated that the residual flavonoids in the JFR could also activate these pathways to increase the proportion of slow-twitch fiber and make the muscle fibers of goats thinner, thus improving the tenderness of meat. Of course, this speculation still needs to be verified through experiments. Therefore, the tenderness of goat meat could be improved by adding JFR to the diet of goats.

The content and types of protein and fatty acids in meat are important factors affecting consumer choice (3). In addition, the composition of amino acids and fatty acids is closely related to the flavor of meat (6). Therefore, we assessed the effect of adding JFR to the diet of goats on the amino acid and fatty acid composition of meat. The addition of JFR increased the content of glutamic acid in goat meat. Glutamic acid is a delicious amino acid, and free glutamic acid is associated with fresh taste (33). This may be one of the main factors for the significant enhancement of the umami taste of goat meat as determined by the electronic tongue system detection. In addition, JFR increased the content of ω-3 PUFAs (ALA and EPA) in goat meat and decreased the ratio of ω-6 to ω-3. ω-3 PUFAs play an important role in the prevention of cardiovascular disease and cancer as well as, in the promotion of vision and neurodevelopment (34, 35). The ratio of ω-6 to ω-3 PUFAs in the diet is closely related to human health. A high ratio of ω-6 to ω-3 PUFAs is associated with cardiovascular diseases, cancer, and other diseases (36). Therefore, JFR improved the nutritional value of mutton by increasing the content of amino acids and ω-3 UFAs in goat meat and reducing the ratio of ω-6 to ω-3 PUFAs.

During the processing of meat products, fat oxidation, Maillard reaction and degradation of flavor precursors will release a large number of volatile components that produce flavor, such as alcohol, aldehyde, carboxylic acid, ester, furan, pyridine, thiophene, nitrogen and sulfur heterocyclic compounds, forming a special flavor of meat (6). Changes in dietary structure can affect the flavor of meat by altering the composition of volatile components (37). For example, the use of *Cistus ladanifer* results in the production of verbenone and 2,2,6-trimethyl-cyclohexanone in mutton (38). In the present study, we found that the use of JFR increased the content of acetaldehyde and hexyl in goat meat. Three volatile components (isovaleraldehyde, 1-octanol, and anethole) were absent in the JFR group, while two alcohols (1-pentanol and 1-pcten-3-pl), one ketone (cyclobutane), four hydrocarbons (hexyl hydride, ethyl acetate, fluorine acetylene, and chlorobenzene), one furan (2-pentylfuran), one amine (octodrine) were detected only in the JFR group. Therefore, the main volatile components of the two groups were different. The main volatile component of the CON group was furan (caramel flavor) (39), while that of the JFR group was aldehydes (fruit aroma) (40). In addition, the volatile components in the feed will also affect the flavor of the meat. Studies have shown that high levels of indole and stercoraline in the feed may enhance the odor of feces and hair in meat (41). There are 71 effective volatiles in the jasmine flower (42). This may be one of the reasons why JFR increased the effective volatile components in the meat of the JFR group. The above results indicate that the addition of JFR to the diet could change the volatile composition of goat meat to affect the odor of goat meat.

Compared with human sensory evaluation, the electronic nose and electronic tongue are more objective (43). They are often used for flavor evaluation and component analysis in the production of foods (44), flowers (45), and pharmaceuticals (46). In meat products, the electronic nose and electronic tongue are often used to evaluate the flavor and freshness (47, 48). In this study, we used the electronic nose and electronic tongue to evaluate the flavor of goat meat. The results of the electronic nose analysis indicated that aromatic components such as nitrogen oxides, short-chain alkanes, inorganic sulfides, alcohols, ethers, aldehydes, ketones, and organic sulfides were significantly increased in the JFR than in the CON group meat. These results are consistent with those of HS-SPME/GC–MS. The taste of meat comes from the flavoring substances in meat, such as inorganic salts, free amino acids, small peptides and nucleic acid metabolites, such as inosinic acid and ribose (49). The results of the electronic tongue analysis indicated that the umami and richness were significantly improved in the JFR than in the CON group meat. This may be due to the increase in glutamic acid content and flavor substances in the JFR group meat. Furthermore, sourness, bitterness, and aftertaste-bitterness and aftertaste-astringency were lower in the JFR than in the CON group meat. Although these several flavors do not appear in meat, this result suggest that the use of JFR in the diet may reduce the impact of compounds that negatively affect the taste of goat meat, improving the flavor of goat meat.

## 5. Conclusion

The present study provides the first evidence that dietary jasmine flower residue supplementation can improve the tenderness and increase the meat content of goat ω- 3 levels of PUFAs while improving the flavor of goat meat. Our study, while unearthing a highly efficient utilization pathway for jasmine flower residue, also provides a reference for animal studies to improve meat quality and flavor by altering the diet structure.

## Data availability statement

The original contributions presented in the study are included in the article/supplementary material, further inquiries can be directed to the corresponding author.

## Ethics statement

The animal study was reviewed and approved by Institutional Animal Care and Use Committee of Guangxi University (GXU2021-902).

## Author contributions

JW, XL, and JZ conceived the study. JW, YL, JL, QL, ZZ, HHa, FD, HHu, HJ, JH, MF, and PX performed the experiments. RL, JW, XY, XL, and JZ analyzed the data and wrote the manuscript. All authors contributed to the article and approved the submitted version.

## Funding

This work was supported by the National key research and development plan “green livable villages and towns technology innovation” key special project “Guangxi Dashi Mountain advantageous characteristic industry quality improvement and efficiency technology integration and demonstration” (2021YFD110010) and the Guangxi Innovation Team Construction Project of National Modern Agricultural Industrial Technology System (nycytxgxcxtd-09-01).

## Conflict of interest

Author FD is employed by Guangxi Nongken Yongxin Animal Husbandry Group Nasuo Animal Husbandry Co., Ltd.

The remaining authors declare that the research was conducted in the absence of any commercial or financial relationships that could be construed as a potential conflict of interest.

## Publisher’s note

All claims expressed in this article are solely those of the authors and do not necessarily represent those of their affiliated organizations, or those of the publisher, the editors and the reviewers. Any product that may be evaluated in this article, or claim that may be made by its manufacturer, is not guaranteed or endorsed by the publisher.
